# Sex differences in the association between metabolic score for insulin resistance and the reversion to normoglycemia in adults with prediabetes: a cohort study

**DOI:** 10.1186/s13098-024-01430-9

**Published:** 2024-07-30

**Authors:** Xiaomin Liang, Zemao Xing, Kai Lai, Xiaohong Li, Shuiqing Gui, Ying Li

**Affiliations:** Department of Critical Care Medicine, Shenzhen Second People’s Hospital, The First Affiliated Hospital of Shenzhen University, Shenzhen, China

**Keywords:** Metabolic score for insulin resistance, Prediabetes, Normoglycemia, Sex differences

## Abstract

**Background:**

The metabolic score for insulin resistance (MetS-IR) has become a valid indicator to evaluate insulin resistance. Our investigation sought gender differences in the correlation between MetS-IR and the reversion from prediabetes to normoglycemic status.

**Methods:**

This retrospective research, carried out in 32 areas across 11 cities with several centers in China, encompassed 15,423 participants with prediabetes. We employed a Cox proportional hazards regression model to examine the link between MetS-IR and the reversion to normoglycemic status. We also applied cubic spline functions and smooth curve fitting to detect non-linear relationships. Additionally, we embarked on a range of sensitivity analyses.

**Results:**

The study included 15,423 participants, with 10,009 males (64.90%) and 5,414 females (35.10%). The average follow-up time was 2.96 ± 0.93 years, and 6,623 individuals (42.94%) reversed normoglycemia. A non-linear correlation was discovered among MetS-IR and reversion to normoglycemic status in men, with a turning point at 55.48. For a one-unit rise in MetS-IR below this point, the chance of reversal to normoglycemic levels declined by 3% (HR = 0.97, 95% CI:0.96–0.97, P *<* 0.0001). In women, the association was linear, with every unit rise in MetS-IR leading to a 3% reduction in transitioning to normal glycemic levels. (HR = 0.97, 95% CI: 0.97–0.98, p *<* 0.0001).

**Conclusion:**

A negative correlation was discovered between MetS-IR and reversion to normoglycemic status in adults with prediabetes. Specifically, a non-linear association was observed for males, while females exhibited a linear correlation.

**Supplementary Information:**

The online version contains supplementary material available at 10.1186/s13098-024-01430-9.

## Introduction

Prediabetes is a bidirectional transition state where glucose metabolism is not normal, but not yet diabetic, encompassing impaired glucose tolerance (IGT) situations as well as impaired fasting glucose (IFG) [1]. Not all prediabetic patients develop into diabetes; some stay prediabetic, and 20–50% may even revert to normoglycemia [2–5]. Prediabetes is a dangerous circumstance that can lead to various chronic and vascular diseases as well as diabetes [1, 6–8]. Recent focus has been on the reversal of prediabetes to normoglycemia, as studies suggested that even a short period of normoglycemia can markedly lower the probability of progressing to diabetes [9]. Reversing prediabetes through medication or lifestyle changes can significantly shield patients from developing diabetes and multiple long-term health issues [10–14]. Considering the worldwide prevalence of prediabetes, with over 400 million individuals affected and facing a broad spectrum of potential health complications [1, 6–8, 15], it’s of utmost importance to investigate changeable elements that can aid in reversing prediabetes.

Insulin resistance (IR) is the diminished role of insulin in regulating glucose absorption and utilization [16], which is a key hazard factor for numerous chronic illnesses [10]. The euglycemic–hyperinsulinemic clamp (EHC), the benchmark for evaluating IR, is an intricate and resource-demanding process that limits its routine clinical implementation [17]. MetS-IR is a novel alternative indicator for evaluating IR and a pragmatic forecaster of diabetes because of its dependability, consistency, and simplicity [17]. MetS-IR was preferred over other surrogate IR indices. Notably, it outperformed non-insulin-based IR indicators like TG/HDL-c and TyG in diagnosing IR and predicting diabetes [17]. Additionally, MetS-IR showed superior efficacy in predicting and diagnosing metabolism-related conditions, including coronary artery disease and prehypertension, surpassing TG/HDL-c and TyG [18, 19]. Moreover, MetS-IR was more accurate than the homeostatic model assessment for IR (HOMA-IR) in predicting the incidence of non-alcoholic fatty liver disease (NAFLD) [20]. Several studies have further confirmed the important role of MetS-IR in prediabetes and diabetes onset [17, 21–24]. Nonetheless, the effect of MetS-IR on the recovery to normoglycemic status in prediabetic individuals remains unclear. Gender differences in the relationship between prediabetes incidents and IR were shown in previous studies [25, 26]. Hence, we hypothesized a gender disparity in the recovery from prediabetes to normal glycemic levels. Given these factors, our investigation attempted to examine the link between MetS-IR and glycemic reversal in prediabetic adults, and the gender differences in these correlations.

## Methods

### Study design and data source

The raw data was freely downloaded from the DATADRYAD database provided by Chen et al [27]. The data originated from a published article titled” Association of body mass index and age with incident diabetes in Chinese adults: a population- based cohort study.” This article is open-access, allowing for remixing, modification, non-commercial sharing, and the creation of derivative works [27]. This study received approval from the Rich Healthcare Group Review Board and the Ethics Committee of Shenzhen Second People’s Hospital (2024-252-01PJ). Given its retrospective nature, the institutional ethics committee waived the requirement for informed consent.

Data were extracted from a computerized database established by the Rich Healthcare Group in China. This database included all medical records for individuals who received medical check-ups across 11 cities and 32 regions from 2010 to 2016. The initial study included 685,277 individuals who were older than 20 and had at least two check-ups. 473,444 excluded individuals met the specific criteria: (1) Missing baseline results of sex, height, fasting plasma glucose (FPG), and weight. (2) Extremely low BMI (*<* 15 kg/m²) and high BMI (*>* 55 kg/m²). (3) Less than 2 years between visits. (4) Baseline diagnosis of diabetes. (5) Uncertain diabetic situation during the entire period. The investigation analyzed data from 211,833 individuals ultimately.

**Fig. 1:**
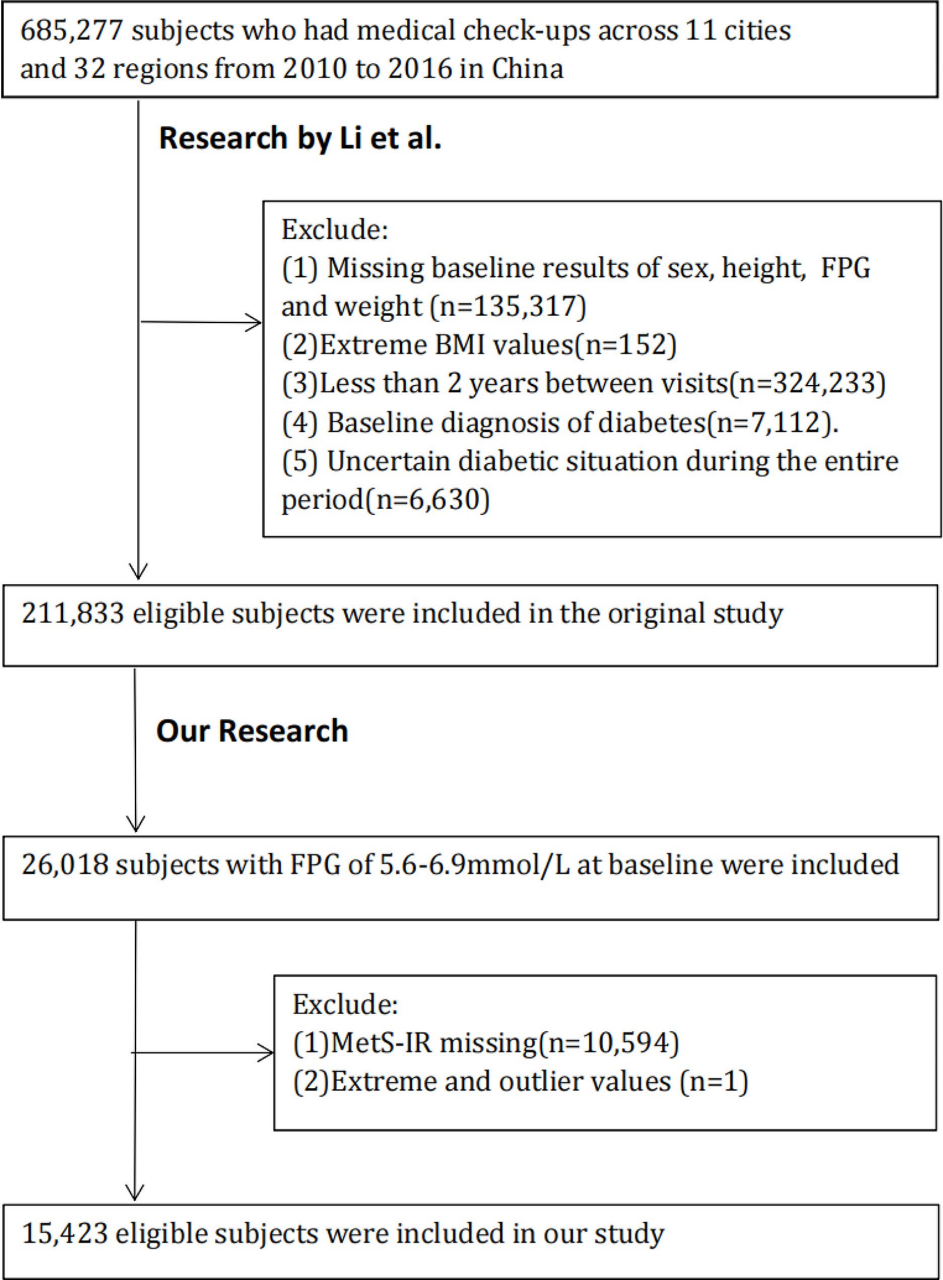
Flow chart of the study population

Based on our investigation design of retrospective cohort study, 26,018 prediabetes individuals were included with FPG baseline results among 5.6 and 6.9 mmol/L, meeting 2021 ADA guidelines standards [28]. Participants with missing baseline data on MetS-IR (10,594 individuals) and those with extreme and outlier values (1 individual) were excluded. The final participant count for our study was 15,423. The flowchart was depicted in Fig. 1.

### Measurement of baseline indicators

During the study, subjects were interviewed and involved in completing a full questionnaire. This questionnaire recorded drinking and smoking habits, past disease history, demographic data, and diabetes genetic history. In a quiet environment, professional staff measured the subjects’ height, Systolic blood pressure (SBP), weight, and Diastolic blood pressure (DBP). For these measurements, individuals were barefoot, dressed in convenient clothing, and had their BP measured using an accurate mercury gauge with a cuff. Subjects should be fasting 10 h before the start of the blood test. Post-collection blood samples were analyzed promptly. Laboratory indicators were treated utilizing automatic analyzing and testing instruments (Beckman 5800), containing Serum creatinine (Scr), low-density lipoprotein cholesterol (LDL-c), triglyceride (TG), aspartate aminotransferase (AST), total cholesterol (TC), blood urea nitrogen (BUN), fasting plasma glucose (FPG), alanine aminotransferase (ALT) and high-density lipoprotein cholesterol (HDL-c).

### Variables

#### Exposure

The Body Mass Index (BMI) is calculated as Weight (kg) / Height (m^2^).

The MetS-IR is calculated as ln [(2×FPG) + TG] ×BMI/ [ln (HDL-c)] [17].

#### Outcome

Our outcome was the reversion to normoglycemia from prediabetes. Prediabetes was determined based on a baseline FPG ranging from 5.6 to 6.9 mmol/L [28]. Reversion to normoglycemia was defined as a final FPG *<* 5.6 mmol/L without self-reported diabetes at the follow-up [5, 28].

### Missing data handling

Missing data were expressed as quantity (percentage). They were BUN (364,2.36%), DBP (5,0.03%), LDL-c (28,0.18%), Drinking status (10676,69.22%), AST (8295,53.78%), SBP (5,0.03%) Scr (116,0.75%), Smoking status (10676,69.22%), ALT (37,0.24%) and Family history of diabetes (401,2.60%). Multiple imputations were applied in the imputation model to deal with missing data to minimize bias which could affect the accurate representation and statistical performance [29, 30]. Scr, Family history of diabetes, Age, Drinking status, SBP, AST, sex, DBP, ALT, Smoking status, LDL-c, and BUN were considered in the imputation model (10 iterations, linear regression). Analyzing missing data process by assuming that the data was missing at random (MAR) [29].

### Statistical analysis

Categorical variables were represented as percentages, while continuous ones as either medians (ranges of interquartile) or as means (standard deviations). The differences following the MetS-IR quartiles were analyzed by applying categorical data with chi-squared tests and continuous data with one-way ANOVA.

Confounding variables were selected according to their association with the outcomes of interest or changes in effect estimates of more than 10% [31]. Confounding variables excluded TC because of the collinearity (Supplementary Table 1). After considering the clinical significance and previous literature, we adjusted for the following covariates: Scr, age, LDL-c, DBP, diabetes family history, ALT, SBP, smoking status, sex, AST, drinking status, and BUN. Three models were developed implementing multivariate Cox proportional hazards regression to seek the relationship between MetS-IR and reversion to normoglycemia in the general population and sex subgroups. Crude model: unadjusted. Model I was adjusted for age and sex. Model II was adjusted for Scr, age, LDL-c, DBP, diabetes family history, ALT, SBP, smoking status, sex, AST, drinking status, and BUN. Sex was not adjusted in gender subgroups. Results were expressed as 95% confidence intervals (95% CI).

The non-linear relationship among MetS-IR and the probability of reversal to normal FPG were further explored in women, men, and total participants. This was done using a Cox proportional hazards regression model with a cubic spline function and smoothed curve fitting. If a non-linear relation was found, a recursive method was first applied to confirm the inflection point. Then a two-piecewise Cox proportional hazards regression model was built on either side of this inflection point. Ultimately, the model that best explained the link between MetS-IR and the reversal to normal FPG was chosen based on a log-likelihood ratio test.

To validate our findings, we implemented several sensitivity analyses. Given the strong association between smoking, hypertension as well as family history of diabetes [32–34], we examined the role of MetS-IR on reversal to the normal FPG incident in men, women, and all participants by smoke-free individuals, non-hypertensive individuals, or those without diabetes family history for the sensitivity analyses. Additionally, we calculated the E-value to determine the necessary magnitude of an unobserved confounder among MetS-IR and reversion from prediabetes to normal FPG [35].

All statistical findings were produced in line with the STROBE declaration [36]. The Statistical findings were carried out by implementing R statistical software tools and Empower Stats. All tests were conducted with a significance level confined to P *<* 0.05.

## Results

### Characteristics

**Table 1:** The baseline and follow-up characteristics of individuals

MetS-IR quartiles	Q1(22.87–38.38)	Q2(38.38–43.27)	Q3(43.27–48.35)	Q4(48.35–86.87)	*P*-value
Participants	3856	3856	3856	3855	
Age(years)	48.82 ± 14.32	51.51 ± 13.24	52.29 ± 12.99	51.10 ± 12.93	< 0.001
DBP (mmHg)	74.63 ± 10.51	77.69 ± 10.74	79.80 ± 10.87	81.90 ± 11.32	< 0.001
BMI (kg/m^2^)	21.07 ± 1.66	23.92 ± 1.35	25.76 ± 1.62	28.58 ± 2.61	< 0.001
SBP (mmHg)	122.28 ± 17.58	126.85 ± 17.50	129.36 ± 17.28	131.52 ± 17.10	< 0.001
FPG (mg/dL)	105.79 ± 4.98	106.78 ± 5.59	107.62 ± 5.88	108.48 ± 6.16	< 0.001
TC (mg/dL)	90.22 ± 17.30	91.12 ± 16.88	91.00 ± 16.86	90.81 ± 17.22	0.094
ALT(U/L)	16.00 (12.10–22.00)	20.40 (15.00–29.00)	24.00 (17.90–35.00)	30.10 (21.00–46.00)	< 0.001
TG (mg/dL)	18.00 (12.78–24.66)	24.12 (17.28–33.84)	29.88 (21.06–41.76)	36.72 (26.10-53.82)	< 0.001
HDL-c (mg/dL)	28.22 ± 5.65	25.05 ± 4.08	22.93 ± 4.07	19.95 ± 4.14	< 0.001
LDL-c (mg/dL)	52.21 ± 13.01	53.34 ± 12.65	53.17 ± 12.71	52.34 ± 13.53	< 0.001
AST(U/L)	23.46 ± 9.86	25.05 ± 10.95	26.37 ± 9.41	29.38 ± 13.73	< 0.001
Scr (µmol/L)	68.18 ± 15.35	72.96 ± 16.49	75.01 ± 15.86	75.99 ± 15.89	< 0.001
BUN (mmol/L)	4.86 ± 1.24	5.04 ± 1.25	5.06 ± 1.22	5.06 ± 1.25	< 0.001
Sex					< 0.001
Male	1729 (44.84%)	2414 (62.60%)	2784 (72.20%)	3082 (79.95%)	
Female	2127 (55.16%)	1442 (37.40%)	1072 (27.80%)	773 (20.05%)	
Family history of diabetes					0.477
No	3769 (97.74%)	3749 (97.23%)	3753 (97.33%)	3751 (97.30%)	
Yes	87 (2.26%)	107 (2.77%)	103 (2.67%)	104 (2.70%)	
Smoking status					< 0.001
Current	169 (15.00%)	251 (22.41%)	342 (27.58%)	451 (35.79%)	
Ever	45 (3.99%)	52 (4.64%)	57 (4.60%)	70 (5.56%)	
Never	913 (81.01%)	817 (72.95%)	841 (67.82%)	739 (58.65%)	
Drinking status					< 0.001
Current	29 (2.57%)	52 (4.64%)	74 (5.97%)	72 (5.71%)	
Ever	165 (14.64%)	234 (20.89%)	268 (21.61%)	275 (21.83%)	
Never	933 (82.79%)	834 (74.46%)	898 (72.42%)	913 (72.46%)	
final FPG (mg/dL)	100.66 ± 13.22	104.75 ± 16.96	106.99 ± 18.61	110.74 ± 23.94	< 0.001
self-reported diabetes					< 0.001
No	3647 (94.58%)	3478 (90.20%)	3345 (86.75%)	3145 (81.58%)	
Yes	209 (5.42%)	378 (9.80%)	511 (13.25%)	710 (18.42%)	
follow-up time(years)	2.82 ± 0.87	2.90 ± 0.90	2.99 ± 0.94	3.11 ± 0.98	< 0.001
Normoglycemia					< 0.001
No	1683 (43.65%)	2169 (56.25%)	2391 (62.01%)	2557 (66.33%)	
Yes	2173 (56.35%)	1687 (43.75%)	1465 (37.99%)	1298 (33.67%)	

Categorical variables were represented as percentages, while continuous ones were either medians (ranges of interquartile) or means (standard deviations)

Table 1 presented the characteristics of the study participants. Our study included 15,423 participants, with a male majority (64.90%) and a female minority (35.10%). The average age was 50.93 ± 13.44, and 6,623 participants (42.94%) returned to normal glucose levels. MetS-IR values ranged from 22.87 to 86.87, averaging 43.69 ± 7.57. MetS-IR was divided into quartiles: Q1 (22.87–38.38), Q2 (38.38–43.27), Q3 (43.27–48.35), and Q4 (48.35–86.87). The male percentage increased from Q1 to Q4, while the female percentage decreased. Values of Age, AST, DBP, ALT, BUN, SBP, Scr, and the percentages of current smokers and drinkers, all showed an increasing trend from Q1 to Q4. The average follow-up time was 2.96 ± 0.93 years. At follow-up, the final FPG level, follow-up time, and self-reported diabetes percentage increased from Q1 to Q4. The incident of returning to normal FPG decreased across the quartiles, with Q1 at 56.35%, Q2 at 43.75%, Q3 at 37.99%, and Q4 at 33.67%. (Table 1)

**Fig. 2:**
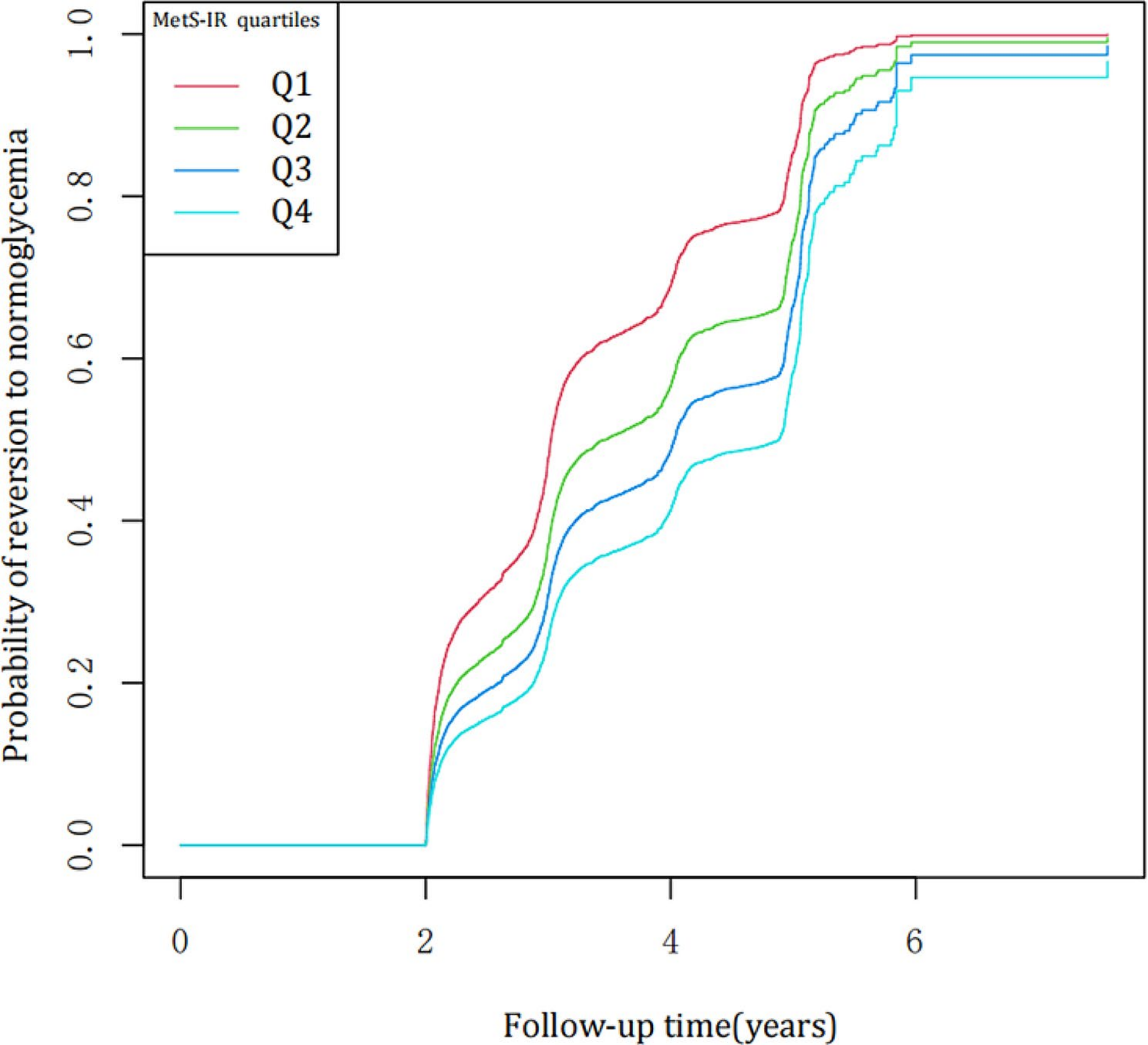
Kaplan-Meier curves for the probability of reversion to normoglycemia from prediabetes

Kaplan-Meier curves revealed that the chances of reversal to normoglycemic levels steadily declined as the MetS-IR rose. This demonstrated that individuals with the largest MetS-IR had the least potential to recover from prediabetes to normoglycemia (Fig. 2).

### Link among MetS-IR and the reversal to normal glycemic levels from prediabetes in all participants

**Table 2:** Relation among MetS-IR and the reversal to normoglycemic levels in male, female, and all participants

Exposure	Crude model (HR, 95%CI, *P*)	Model I (HR, 95%CI, *P*)	Model II (HR, 95%CI, *P*)
**Total**	0.96 (0.96, 0.97) < 0.0001	0.97 (0.96, 0.97) < 0.0001	0.97 (0.97, 0.97) < 0.0001
MetS-IR quartiles			
Q1	1.0	1.0	1.0
Q2	0.72 (0.68, 0.77) < 0.0001	0.78 (0.73, 0.83) < 0.0001	0.80 (0.75, 0.86) < 0.0001
Q3	0.58 (0.54, 0.62) < 0.0001	0.64 (0.60, 0.69) < 0.0001	0.67 (0.63, 0.72) < 0.0001
Q4	0.46 (0.43, 0.50) < 0.0001	0.50 (0.47, 0.54) < 0.0001	0.55 (0.51, 0.59) < 0.0001
P for trend	< 0.0001	< 0.0001	< 0.0001
**Male**			
MetS-IR	0.96 (0.96, 0.97) < 0.0001	0.96 (0.96, 0.97) < 0.0001	0.97 (0.96, 0.97) < 0.0001
MetS-IR quartiles			
Q1	1.0	1.0	1.0
Q2	0.75 (0.68, 0.82) < 0.0001	0.79 (0.72, 0.87) < 0.0001	0.81 (0.74, 0.89) < 0.0001
Q3	0.58 (0.53, 0.64) < 0.0001	0.64 (0.58, 0.70) < 0.0001	0.67 (0.61, 0.73) < 0.0001
Q4	0.46 (0.42, 0.51) < 0.0001	0.49 (0.45, 0.53) < 0.0001	0.53 (0.49, 0.59) < 0.0001
P for trend	< 0.0001	< 0.0001	< 0.0001
**Female**			
MetS-IR	0.96 (0.95, 0.97) < 0.0001	0.97 (0.96, 0.97) < 0.0001	0.97 (0.97, 0.98) < 0.0001
MetS-IR quartiles			
Q1	1.0	1.0	1.0
Q2	0.68 (0.62, 0.75) < 0.0001	0.75 (0.68, 0.82) < 0.0001	0.78 (0.70, 0.85) < 0.0001
Q3	0.58 (0.52, 0.64) < 0.0001	0.64 (0.58, 0.72) < 0.0001	0.68 (0.61, 0.77) < 0.0001
Q4	0.49 (0.43, 0.56) < 0.0001	0.54 (0.48, 0.62) < 0.0001	0.60 (0.52, 0.69) < 0.0001
P for trend	< 0.0001	< 0.0001	< 0.0001

Crude model: unadjusted

Model I was adjusted for age and sex

Model II was adjusted for Scr, age, LDL-c, DBP, diabetes family history, ALT, SBP, smoking status, sex, AST, drinking status, and BUN.

Sex was not adjusted in gender subgroups

To seek the relation between MetS-IR and the reversal to normal glycemic levels, we constructed three Cox proportional hazards regression models across all participants (Table 2). In the crude model, per-unit rise in MetS-IR related to a 4% reduction in the reversal incident (HR = 0.96, 95% CI: 0.96–0.97). In Model I and II, per-unit enhancement in MetS-IR led to a 3% decline in this reversal incident (HR = 0.97, 95% CI: 0.96–0.97 and HR = 0.97, 95%CI: 0.97–0.97, respectively). Moreover, we reintegrated MetS-IR, transformed into categorical variables, into the model. Compared to Q1 participants, the multivariate-adjusted model showed HRs of 0.80 (95% CI: 0.75–0.86) for Q2, 0.67 (95% CI: 0.63–0.72) for Q3, and 0.55 (95% CI: 0.51–0.59) for Q4 participants (Table 2).

### Relation among MetS-IR and the reversal to normoglycemic levels from prediabetes stratified by gender

We utilized Cox proportional hazards regression models to study the role of MetSIR and the reversal to normal FPG in both genders. The multivariate-adjusted model showed that for male subjects, a one-unit enhancement in MetS-IR brought about a 3% decline in the chance of reverting to normal FPG (HR = 0.97, 95%CI: 0.96–0.97). Compared to Q1 participants, the HRs for Q2, Q3, and Q4 were 0.81 (95%CI: 0.74-0.89), 0.67 (95%CI: 0.61–0.73), and 0.53 (95%CI: 0.49–0.59), respectively. (Table 2). For female subjects, per unit rise in MetS-IR was connected with a 3% reduction in the reversal incident (HR = 0.97, 95% CI: 0.97–0.98). Relative to Q1 participants, the HRs for Q2, Q3, and Q4 were 0.78 (95%CI: 0.70–0.85), 0.68 (95%CI: 0.61–0.77), and 0.60 (95%CI: 0.52–0.69), respectively. (Table 2).

### Examining non-linear associations with Cox proportional hazards regression and cubic spline functions, as well as smooth curve fitting

**Fig. 3:**
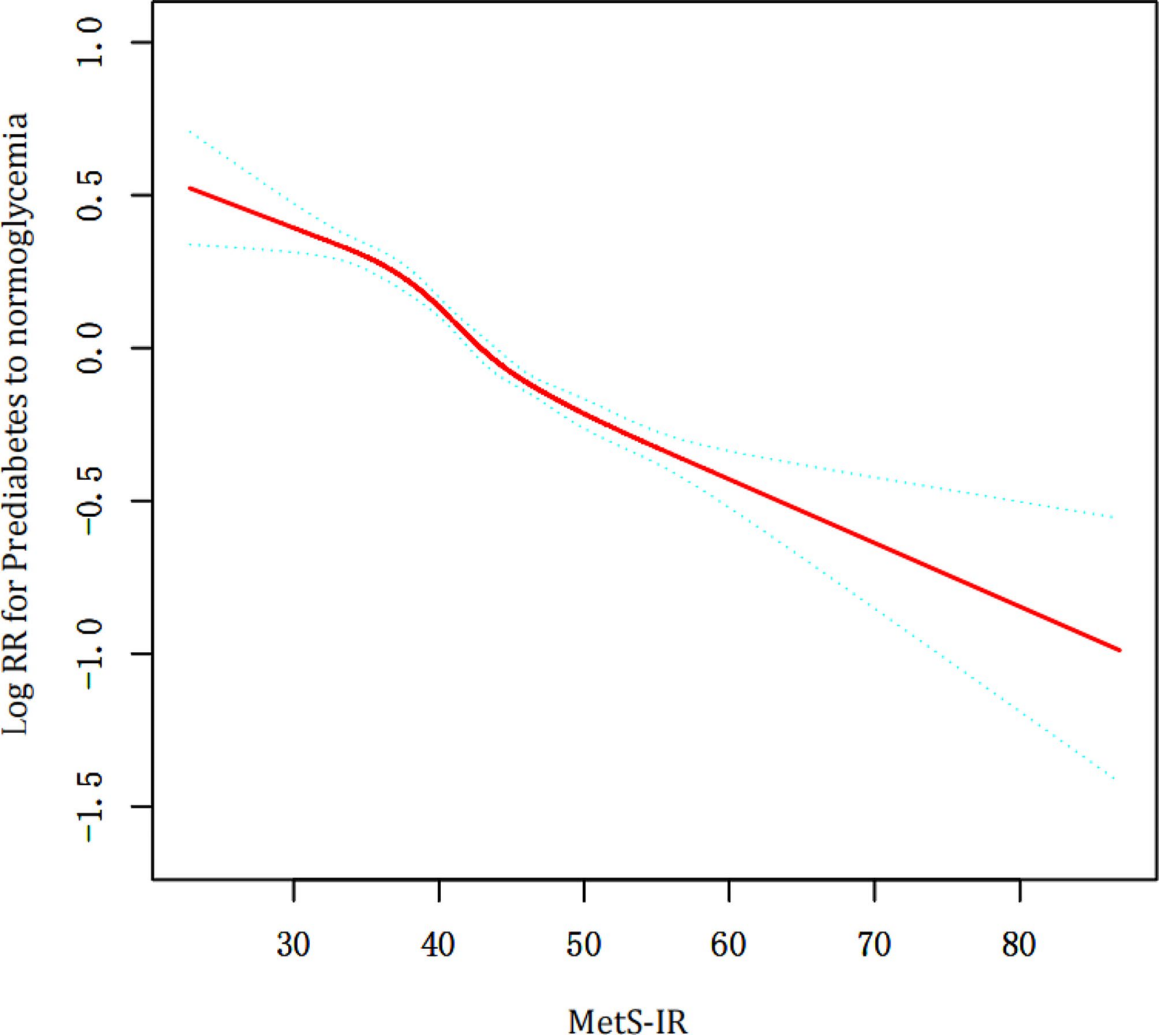
The non-linear relation among MetS-IR and the reversal to normoglycemic levels from prediabetes in all participants

**Table 3:** The results of the two-piecewise Cox regression model

	Male (HR, 95%CI, *P*)	Female (HR, 95%CI, *P*)	Total (HR, 95%CI, *P*)
Standard Cox regression	0.97 (0.96, 0.97) < 0.0001	0.97 (0.97, 0.98) < 0.0001	0.97 (0.97, 0.97) < 0.0001
Two-piecewise Cox regression			
Inflection points of MetS-IR	55.48	43.63	55.47
≤ Inflection point	0.97 (0.96, 0.97) < 0.0001	0.97 (0.96, 0.98) < 0.0001	0.97 (0.96, 0.97) < 0.0001
> Inflection point	1.00 (0.98, 1.02) 0.8817	0.98 (0.97, 0.99) 0.0041	0.99 (0.98, 1.01) 0.4329
P for log-likelihood ratio test	0.003	0.162	0.011

Note 1: Adjusted for Scr, age, DBP, diabetes family history, sex, ALT, smoking status, SBP, drinking status, AST, LDL-c, and BUN.

Note 2: Sex was not adjusted in gender subgroups

Our study found a non-linear relation between MetS-IR and the reversal to normoglycemic levels (Fig. 3; Table 3). The turning point in this relationship occurred at a MetS-IR value of 55.47. For each unit rise in MetS-IR below this point, the chance of the reversal to normoglycemic status declined by 3% (HR = 0.97, 95% CI: 0.96–0.97, p *<* 0.0001). Above this inflection point of 55.47, the HR was 0.99 with a 95% CI (0.98-1.01), indicating no significant change in the likelihood of reversal to normoglycemic status (Table 3).

### Non-linear relation among MetS-IR and the likelihood of reversal to normal glycemic levels in gender subgroups

**Fig. 4:**
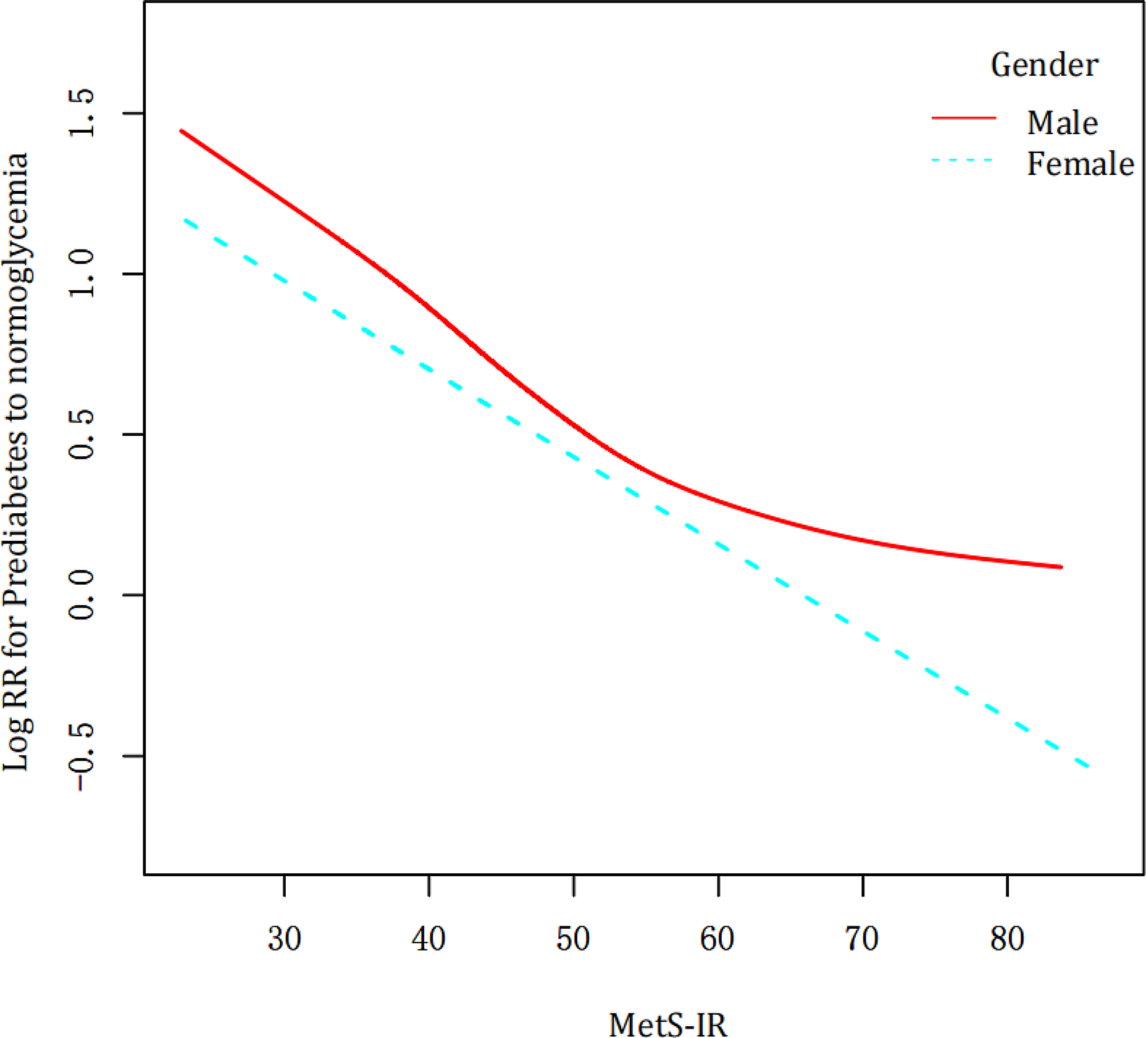
The non-linear relation among MetS-IR and the likelihood of reversal to normal glycemic levels in gender subgroups

Our study found a non-linear relation among MetS-IR and reversal to normal FPG in males (Fig. 4; Table 3). The turning point in this relationship occurred at a MetS-IR value of 55.48. For each unit rise in MetS-IR below this point, the chance of reversing declined by 3% (HR = 0.97, 95% CI:0.96–0.97, P<0.0001). Above this inflection point, the relationship was not obvious among males. However, this non-linear relationship did not exist in women (Fig. 4; Table 3).

### Sensitivity analysis

We conducted four sensitivity analyses to validate our results: Model I included subjects without diabetes family history; Model II included subjects with SBP under 140mmHg; Model III included subjects with DBP under 90mmHg; Model IV included non-smokers. All models confirmed the main findings of a non-linear relationship in men, but not in women, demonstrating the robustness of our investigation (Table 4).

**Table 4:** Association among MetS-IR and the reversion from prediabetes to normoglycemic levels in different sensitivity analyses

	Male (HR, 95%CI, *P*)	Female (HR, 95%CI, *P*)	Total (HR, 95%CI, *P*)
**Model I**			
Standard Cox regression	0.97 (0.96, 0.97) < 0.0001	0.97 (0.97, 0.98) < 0.0001	0.97 (0.97, 0.97) < 0.0001
Two-piecewise Cox regression			
Inflection points of MetS-IR	55.51	43.62	55.49
≤ Inflection point	0.96 (0.96, 0.97) < 0.0001	0.97 (0.96, 0.98) < 0.0001	0.97 (0.96, 0.97) < 0.0001
> Inflection point	1.00 (0.98, 1.02) 0.8931	0.98 (0.97, 0.99) 0.0049	0.99 (0.97, 1.01) 0.3766
P for log-likelihood ratio test	0.003	0.185	0.016
**Model II**			
Standard Cox regression	0.97 (0.96, 0.97) < 0.0001	0.97 (0.96, 0.98) < 0.0001	0.97 (0.96, 0.97) < 0.0001
Two-piecewise Cox regression			
Inflection points of MetS-IR	53.17	44.23	52.86
≤ Inflection point	0.96 (0.96, 0.97) < 0.0001	0.96 (0.96, 0.97) < 0.0001	0.97 (0.96, 0.97) < 0.0001
> Inflection point	0.99 (0.97, 1.01) 0.2127	0.98 (0.97, 1.00) 0.0462	0.98 (0.97, 1.00) 0.0676
P for log-likelihood ratio test	0.029	0.081	0.045
**Model III**			
Standard Cox regression	0.97 (0.96, 0.97) < 0.0001	0.97 (0.96, 0.98) < 0.0001	0.97 (0.96, 0.97) < 0.0001
Two-piecewise Cox regression			
Inflection points of MetS-IR	54.97	43.64	55.05
≤ Inflection point	0.96 (0.96, 0.97) < 0.0001	0.97 (0.96, 0.98) < 0.0001	0.97 (0.96, 0.97) < 0.0001
> Inflection point	1.00 (0.98, 1.02) 0.8766	0.98 (0.96, 0.99) 0.0048	0.99 (0.97, 1.01) 0.3699
P for log-likelihood ratio test	0.004	0.254	0.021
**Model IV**			
Standard Cox regression	0.97 (0.96, 0.98) < 0.0001	0.97 (0.97, 0.98) < 0.0001	0.97 (0.97, 0.98) < 0.0001
Two-piecewise Cox regression			
Inflection points of MetS-IR	57.63	43.64	56.29
≤ Inflection point	0.97 (0.96, 0.97) < 0.0001	0.97 (0.96, 0.98) < 0.0001	0.97 (0.97, 0.97) < 0.0001
> Inflection point	1.00 (0.97, 1.04) 0.8214	0.98 (0.97, 0.99) 0.0038	0.99 (0.97, 1.01) 0.4238
P for log-likelihood ratio test	0.045	0.176	0.116

Model I: individuals without a diabetes family history. Adjustment with Scr, age, DBP, BUN, sex, ALT, Drinking status, SBP, smoking status, AST, and LDL-c.

Model II: individuals with SBP below 140mmHg. Adjustment with Scr, age, DBP, diabetes family history, sex, ALT, smoking status, Drinking status, AST, LDL-c, and BUN.

Model III: individuals with DBP below 90mmHg. Adjustment with Scr, age, SBP, diabetes family history, sex, ALT, smoking status, Drinking status, AST, LDL-c, and BUN.

Model IV: non-smokers. Adjustment with age, Scr, DBP, LDL-c, sex, ALT, Drinking status, SBP, diabetes family history, AST, and BUN.

Note 2: Adjustment without sex in gender subgroups

We used E-values to quantify the robustness of possible unmeasured confounders in the findings. Our results remained solid unless an unmeasured confounder with an HR exceeding 1.17.

## Discussion

In the comprehensive longitudinal research on a Chinese demographic, we found a negative relation among MetS-IR and the reversal from prediabetes to normal glycemic levels in all genders. Our research revealed a non-linear relation with a saturation effect among MetS-IR and the recovery from prediabetes to normal FPG in men, with a turning point at a MetS-IR of 55.48. In contrast, for women, there was a linear relationship. To our knowledge, this may be the first investigation to underscore the connection between gender differences and the likelihood of reversing prediabetes through MetS-IR.

Prediabetes is an intermediate transition state that can either return to normal or deteriorate into diabetes mellitus [1]. Prediabetes can both deteriorate into diabetes or attack organ systems throughout the body, involving the kidney, cerebrovascular and cognitive ability, lung, cardiovascular, and liver, ultimately affecting survival and quality of life with long-term chronic disease [1, 10–14]. It is reassuring and promising that the high blood sugar condition of prediabetes can be reversed. Malm¨o Feasibility Study suggested that more than half of the prediabetic patients reverted to normal glucose levels within 6 years through lifestyle interventions such as diet and exercise [37]. The Whitehall II cohort study indicated that 45% of IFG people achieved normal levels within five years [38]. Canadian STOP-NIDDM cohort showed that 35% of IGT patients returned to normal glucose within 1300 days of treatment with acarbose [39]. These various studies conducted globally, whether on pharmacological or lifestyle interventions, exemplify the likelihood and reliability of transitioning from the early warning period of pre-diabetes to the safe period of normal blood glucose. In our recent study, over a mean period of 2.9 years, we found that 42.94% of prediabetic subjects normalized their blood glucose levels. This outcome aligned closely with the prediabetes reversal rate reported in the aforementioned study.

The gender differences in the relation among MetS-IR and the transition from prediabetes to normoglycemic status found in our present investigation could be partly supported by three facts. First, it was related to the condition that most of the females in this study were of perimenopausal age (mean age 51). A cohort of 37,317 participants showed that men had the fastest increase in lipids before age 40, while women experienced a “spike” in lipids between the ages 40 and 49 [40]. Estrogen plays an important role in regulating blood lipids and IR [41]. Women before menopause had a lower incidence of IR compared to men of the same age, and postmenopausal women had increased odds of developing diabetes [42]. The disappearance of the protective effect of estrogen leading to a drastically increased probability of diabetes might explain the absence of threshold and saturation effects of conversion to normoglycemia from prediabetes and MetS-IR, but rather a linear relationship. Second, gender differences exist in body fat distribution and activity levels. Typically, women carry more body fat and engage in less activity than men [43, 44]. The adverse effects of glucose metabolism and fat deposition lead to IR in organs that are not predominantly fatty [45]. Higher fat content in women would raise diabetes incidents. Moreover, physical exercise may speed up glucose production and metabolism through a series of intricate processes [46]. Women’s comparatively lower physical activity levels may hinder glucose metabolism, leading to high glycemic levels.

Given the above analysis, to achieve the same reversal rate, women would need a lower MetS-IR, implying that managing MetS-IR in women would necessitate more rigorous and earlier interventions. For men, keeping MetS-IR below 55.48 significantly increases the likelihood of reversing prediabetes to normal glucose levels. However, when MetS-IR exceeds 55.48 in men, a saturation effect occurs, and simply lowering MetS-IR won’t significantly increase the chances of reversing prediabetes. At this point, it may be necessary to manage other hazard factors, such as smoking and hypertension.

### Study strengths and limitations

It’s noteworthy that: (1) This investigation may be the initial proposal to clarify the relation among MetS-IR and the reversal from prediabetes to normoglycemic status. (2) After establishing the negative relation among MetS-IR and the reversal to normal FPG from prediabetes, this study further discovered gender differences in this relationship. (3) For men, the relation among MetS-IR and the reversal to normal FPG from prediabetes was non-linear, with inflection points identified. (4) Given the large sample population and multi-center approach of this investigation, the evidence it provides can be considered quite robust.

This study also has limitations: (1) The investigation is launched on the Chinese population so conclusions are not suitably focused on other racial groups. (2) The definition of prediabetes lacks a standard of glycated hemoglobin levels or IGT. (3) The investigation is a secondary analysis of existing data and, hence, lacks dietary and exercise-related metrics. Although the E-value is used to measure the effect of potential hidden confounders, it is less likely to fully explain the observed findings of these confounders.

## Conclusion

In a longitudinal and cohort study involving the Chinese population, we found that MetS-IR was inversely associated with reversion from prediabetes to normoglycemia. Furthermore, males exhibited a non-linear relationship, while females showed a linear association. Our study emphasized the pivotal role of MetS-IR in assessing prediabetes reversion from a therapeutic perspective. These insights provide a valuable reference for managing prediabetes, especially considering gender differences. For females to manage MetS-IR, rigorous early interventions are necessary, whereas males should maintain MetS-IR below 55.48 for successful reversion to normoglycemia. When MetS-IR exceeds this threshold, addressing other risk factors (such as smoking and hypertension) becomes essential. Future ethnically diverse and multicenter studies are needed to validate our findings.

### Electronic supplementary material

Below is the link to the electronic supplementary material.

Supplementary Material 1

## Data Availability

Data can be downloaded for free from the “DATADRYAD” database (www.Datadryad.org).

## Abbreviations

MetS-IR: Metabolic score for insulin resistance
IR: Insulin resistance
IGT: Impaired glucose tolerance
IFG: Impaired fasting glucose
SBP: Systolic blood pressure
DBP: Diastolic blood pressure
BMI: Body mass index
ALT: Alanine aminotransferase
AST: Aspartate aminotransferase
HDL-c: High-density lipoprotein cholesterol
TC: Total cholesterol
TG: Triglyceride
LDL-c: Low-density lipoprotein cholesterol
FPG: Fasting plasma glucose
HR: Hazard ratio
CI: Confidence interval
Scr: Serum creatinine
BUN: Blood urea nitrogen
