# Supplementary material for: Sex differences in the association between metabolic score for insulin resistance and the reversion to normoglycemia in adults with prediabetes: a cohort study
Source: Diabetol Metab Syndr. 2024 Jul 30;16:183. doi: 10.1186/s13098-024-01430-9 (PMC11288094; doi:10.1186/s13098-024-01430-9)
Supplement: Supplementary file 1 — Supplementary Material 1 [file 13098_2024_1430_MOESM1_ESM.docx]

Sex differences in the association between metabolic score for insulin resistance and the

reversion to normoglycemia in adults with prediabetes: a cohort study

Xiaomin Liang^1^, Zemao Xing^1^, Kai Lai^1^, Xiaohong Li^1^,

Shuiqing Gui^1*^*^†^*, Ying Li^1*^*^†^*

^1^Department of Critical Care Medicine, Shenzhen Second People’s Hospital, The First Affiliated Hospital of Shenzhen University, Shenzhen, China.

*Corresponding author(s). E-mail(s): guishuiqing@163.com; liying20020813@163.com;

Contributing authors: 171283296@qq.com; zemaox@126.com; lk_fe@163.com; 26388956@*qq.com*;

*^†^*These authors contributed equally to this work.

Supplementary Table 1: Collinearity diagnostics steps.

|  | Variance inflation factor | | | |
| --- | --- | --- | --- | --- |
|  | Step 1 | Step 2 | Step 3 | Step 4 |
| MetS-IR | 45.5 | 45 | 2.8 | 2.8 |
| Age | 1.3 | 1.3 | 1.3 | 1.3 |
| Sex | 3.5 | 3.4 | 3.4 | 3.4 |
| Height | 54.3 | 2 | 2 | 2 |
| Weight | 180.5 | NA | NA | NA |
| BMI | 119.2 | 24.1 | NA | NA |
| SBP | 2 | 2 | 2 | 2 |
| DBP | 2 | 2 | 2 | 2 |
| FPG | 1.1 | 1.1 | 1.1 | 1.1 |
| TC | 6.6 | 6.6 | 6.6 | NA |
| LDL-c | 5.5 | 5.5 | 5.5 | 1.1 |
| ALT | 3.4 | 3.4 | 3.4 | 3.4 |
| AST | 3 | 3 | 3 | 3 |
| BUN | 1.2 | 1.2 | 1.2 | 1.2 |
| Scr | 2.2 | 2.2 | 2.2 | 2.2 |
| Family history of diabetes | 1.3 | 1.3 | 1.3 | 1.3 |
| Smoking status | 1.2 | 1.2 | 1.2 | 1.2 |
| Drinking status | 1 | 1 | 1 | 1 |

Note-1: Variance inflation factor = 1/(1-R^2^).

Note-2: The variables with Variance inflation factor >5 will be regarded as collinear variables.

Confounding variables excluded TC because of the collinearity (Supplementary Table 1).

The exposure and outcome variables in the paper include FPG, which is analyzed as an adjusted confounding variable considering circular reasoning and potential bias. Below are the results of comparative analyses of the core results, both without and with the addition of the FPG adjustment variable:

Supplementary Table 2：The results of the two-piecewise Cox regression model

|  | Male (HR, 95%CI, P) | Female (HR, 95%CI, P) | Total (HR, 95%CI, P) |
| --- | --- | --- | --- |
| Standard Cox regression | 0.97 (0.97, 0.98) <0.0001 | 0.98 (0.97, 0.98) <0.0001 | 0.98 (0.97, 0.98) <0.0001 |
| Two-piecewise Cox regression |  |  |  |
| Inflection points of MetS-IR | 55.47 | 43.97 | 55.23 |
| ≤ Inflection point | 0.97 (0.97, 0.98) <0.0001 | 0.97 (0.96, 0.98) <0.0001 | 0.97 (0.97, 0.98) <0.0001 |
| > Inflection point | 1.01 (0.99, 1.02) 0.5853 | 0.98 (0.97, 1.00) 0.0215 | 1.00 (0.98, 1.02) 0.8286 |
| P for log-likelihood ratio test | 0.003 | 0.163 | 0.009 |

Note 1: Adjusted for Scr, age, DBP, diabetes family history, sex, ALT, smoking status, SBP, drinking status, AST, LDL-c, BUN, and FPG.

Note 2: Sex was not adjusted in gender subgroups.

From Table S2, we can see that the core results adjusted for the addition of the FPG variable are still non-linearly related to MetS-IR and reversal from prediabetes to normoglycemia in men compared to not adding an adjustment, with a similar inflection point (55.47 vs. 55.48) and a significant negative correlation (HR = 0.97, 95% CI: 0.97–0.98, P<0.0001) versus (HR = 0.97, 95% CI: 0.96–0.96, P<0.0001). In women, the relationship remained linear (HR = 0.98, 95% CI: 0.97–0.98, p<0.0001) versus (HR = 0.97, 95% CI: 0.97–0.98, p<0.0001).
